# Semi Salix Leaf Textured Gas Mechanical Face Seal with Enhanced Opening Performance

**DOI:** 10.3390/ma14247522

**Published:** 2021-12-08

**Authors:** Linqing Bai, Pengcheng Zhang, Zulfiqar Ahmad Khan

**Affiliations:** 1 Key Lab of Industrial Fluid Energy Conservation and Pollution Control, Ministry of Education, Qingdao University of Technology, Qingdao 266520, China; 2 School of Mechanical and Automotive Engineering, Qingdao University of Technology, Qingdao 266520, China; zhangpengcheng6538@163.com; 3 NanoCorr, Energy & Modeling (NCEM) Research Group, Department of Design and Engineering, Bournemouth University, Dorset BH12 5BB, UK; ZKhan@bournemouth.ac.uk

**Keywords:** semi salix leaf textures, gas mechanical face seal, open force, leakage, friction torque

## Abstract

Seal performance of a novel gas mechanical face seal with semi salix leaf textures was introduced and theoretically investigated with the purpose of enhancing hydrostatic and hydrodynamic opening performance. First, a theoretical model of a laser surface textured gas mechanical face seal with semi salix leaf textures was developed. Second, the impact of operating and texturing parameters on open force, leakage, and friction torque was numerically investigated and has been discussed based on gas lubrication theory. Numerical results demonstrate that the semi salix leaf textured gas face seal has larger hydrostatic and hydrodynamic effects than the semi ellipse textured seal because of the effect of the inlet groove. All semi salix leaf textured surfaces had better open performance than the semi ellipse textured surface, which means that the inlet groove plays an important role in improving open performance and consequently decreasing contact friction during the start-up stage. Texturing parameters also influenced the seal performance of thee semi salix leaf textured gas face seal. When the inclination angle was 50°, the radial proportion of the inlet groove was 0.8, the dimple number was 9, and the open force resulted in the maximum value. This research has demonstrated the positive effects of the applications of a semi salix leaf textured gas mechanical face seal that combines the excellent hydrostatic and hydrodynamic effects of groove texture and the excellent wear resistance of microporous textures.

## 1. Introduction

A dry gas face seal is a kind of non-contact mechanical seal, which obtains a sealing effect by a gas sealing gas or gas sealing liquid [1]. Due to its outstanding advantages such as low power consumption, long working life, and low leakage rate, dry gas seals have been applied more and more widely in the compressors of pump stations with high-speed turbine compressors and natural gas pipelines [2]. However, a dry gas seal is prone to contact and rapid wear under start-up, shutdown, and transient change conditions, which leads to seal performance decline or even failure. Therefore, it is urgent to develop new theory and technology to make dry gas seals have better lifting ability and maintain seal stability.

The rapid development of surface texturing technology [3,4,5,6] provides an effective way to improve the friction characteristics of the sealing face. Micro-pored structure and the distribution of laser surface texturing in a seal ring play an important role in terms of mechanical face seal performance, especially the open force, friction torque, and leakage, which significantly influence the seal performance. The first theoretical analysis model of a circle micro-pored mechanical face seal was established and then a series of theoretical and experimental studies on circle micro-pored liquid mechanical face seals were conducted by Etsion’s group and other scholars [7,8,9,10,11,12,13,14,15,16]. Etsion and Yu’s research has shown that circle micro-pored mechanical face seals could reduce friction torque and temperature [8,9,10]. Further study by Etsion and Song showed that a partial micro-pored mechanical face seal had better seal performance [11,12]. The circle micro-pored structure also had a better seal performance when being used in a gas seal, however, the hydrodynamic performance fell below threshold requirements when it was applied in liquid, especially in a high pressure condition [13,14,15,16]. In order to improve the hydrodynamic effect and seal performance, Peng discussed the micro-pored geometric structure effect on seal performance. Numerical analysis has shown that a mechanical seal face with either a column or rectangle section profile of micro-pores exhibited the best performance [17]. Bai and Li investigated the hydrodynamic effect of a single-row inclination ellipse dimple gas seal [18,19,20]. Theoretical and experimental work demonstrated that single-row inclination ellipse dimples had a more significant hydrodynamic effect than the circle dimples.

Though theoretical and experimental research revealed that micro dimple textured face seals can provide better friction performance than the un-textured face seal, the opening performance, which related to friction during startup and frictional reliability, was still inferior to radial groove textured seals because substantial hydrodynamic pressure can be generated over the alternating land and groove configuration [21,22].

A novel elliptical dimple gas seal with an inlet groove, which has been named as a semi salix leaf textured gas mechanical face seal, was introduced and theoretically studied in this research. The new seal will combine the excellent hydrostatic and hydrodynamic effects of groove texture and the excellent wear resistance of microporous textures. At first, the conventional calculation based theoretical model of a semi salix leaf textured gas seal was developed, which is based on gas lubrication theory. Then, the seal performance of open force, leakage, and friction torque were calculated and analyzed under various operating conditions including rotational speed, seal clearance, and pressure. Finally, the influence of texturing parameters on seal performance were studied.

This research provided a valuable parametric design method applied to the semi salix leaf texture gas mechanical face seal with enhanced opening performance, which will decrease the risk of contact and wear during start-up as much as possible.

## 2. Theoretical Model

### 2.1. Semi Salix Leaf Textured Gas Mechanical Seal

**Figure 1:**
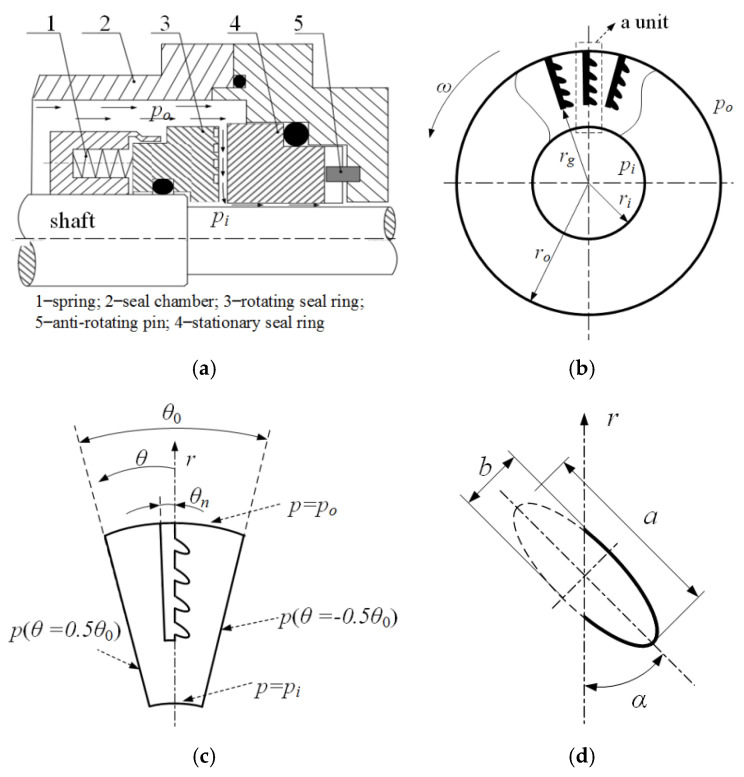
Geometrical model of the seal face with semi salix leaf textures: (**a**) diagram of gas seal, (**b**) textured rotating seal ring, (**c**) one periodic textured unit, (**d**) a micro dimple.

To analyze seal performance of semi salix leaf textured gas mechanical face seal, theoretical analysis was conducted in this research. Figure 1 displays the geometrical model of laser textured mechanical face seal with semi salix leaf textures. As shown in Figure 1a, the mechanical face seal was mainly composed of seal chamber, rotating seal ring, stationary seal ring, spring, and anti-rotating pin. When two seal surfaces were static, the stationary ring and the rotating ring were bonded together under the spring force to prevent medium leakage along the seal end face. If the shaft rotated, the rotating ring also rotated with the shaft and the stationary ring was kept still under the action of anti-rotating pin. Textures were distributed on the rotating ring or the stationary ring seal rings. When two sealing surfaces rotated relatively, a gas film with certain thickness was formed between two surfaces and formed opening force. When the opening force was equal to the closing force formed by the spring and the medium, two seal faces kept a stable operation condition to prevent seal medium leakage along the end face. The opening force was obtained by integrating the gas film pressure.

As shown in Figure 1b, the textures were distributed uniformly along the circumference between the inside seal radius *r_i_* and outside seal radius *r_o_*. The texture consists of two connected parts including a gas inlet groove and several semi-elliptical micro dimples (see Figure 1b). The gas inlet groove with a circumferential angle of *θ_n_* and depth of hg was distributed radially between *r_o_* and *r_g_*. *n_r_* is the semi-elliptical dimple numbers in the radial direction (see Figure 1c, *n_r_* = 4). A spherical segment with a long axial radius *a*, short axial radius *b*, dimple depth *h_d_*, and angle of inclination *α* was used to model each micro semi salix leaf dimple (see Figure 1d). To incorporate the effect of micro-dimple orientation, a slender ratio *γ* was defined as below.

(1) $$\gamma =\frac{a}{b},$$

The incline angle *α* reflects the inclined direction of the semi-elliptical micro dimples. When *γ* is one, it indicates that the micro-dimples are semi-circular.

### 2.2. Mathematical Model

The pressure distribution can be obtained by solving Reynolds governing equations with boundary conditions. Then, the opening force of the entire seal end face can be obtained by integrating the gas film pressure.

#### 2.2.1. Governing Equation and Boundary Condition

(1) Gas film governing equation

Theoretical gas pressure distribution can be acquired by solving the following ideal Reynolds equation for Newtonian compressible fluid with the finite difference method [23].

(2) $$\frac{\partial }{r\partial \theta }\left(\frac{h^{3}}{\eta }\frac{\partial p^{2}}{r\partial \theta }\right)+\frac{\partial }{\partial r}\left(\frac{h^{3}}{\eta }\frac{\partial p^{2}}{\partial r}\right)=12\omega \frac{\partial \left(ph\right)}{\partial \theta },$$

where *θ* and *r* represent the circumferential and radial coordinates, respectively, and *p* is the pressure on the seal face, while *h* is the gas film thickness between seal faces, *η* is gas viscosity, and *ω* is the seal ring speed.

(2) Film thickness equation

For representing thickness of gas film, *c* is defined as the gas film thickness in non-textured area between the two seal surfaces, *h_d_* is the depth of micro-dimples, and *h_g_* is the depth of inlet groove. The governing equation of the gas film thickness at any point between the end faces can be described as follows.

(3) $$h(r,\theta )=\left\{ \begin{array}{l} c,\\ c+h_{d},\\ c+h_{g}, \end{array}\right.\begin{array}{c} \mathrm{un}-\mathrm{textured}\;\mathrm{zone}\\ \mathrm{micro}\;\mathrm{dimples}\\ \mathrm{inlet}\;\mathrm{groove} \end{array},$$

(3) Boundary conditions

The model has two types of boundary conditions, one is mandatory boundary condition and the other is periodic boundary condition.

a. Mandatory boundary condition

(4) $$p(r=r_{i},\theta )=p_{i},$$

(5) $$p(r=r_{o},\theta )=p_{o},$$

In the formula, *p* represents the pressure between the seal surfaces, *p_o_* is the outside pressure at the seal face radius *r_o_*, and *p_i_* is the inner pressure at the radius of the inner diameter *r_i_*.

b. Periodic boundary condition

As the textures are distributed periodically along the circumference, the research objective only takes one unit. Then, the parameters for the entire seal face can be obtained by multiplying the number of periods *N*. The periodic boundary conditions for each unit can be described as follows.

(6) $$p(r,\theta =0.5\theta_{0})=p(r,\theta =-0.5\theta_{0}),$$

#### 2.2.2. Parameters of Seal Performance

The opening force can be deduced by the integration of pressure over the seal areas and is given as below.

(7) $$\bar{F_{o}}=\int_{0}^{2\pi }\int_{r_{i}}^{r_{o}}prdrd\theta ,$$

The leakage rate is in the radial direction. As stated by the flow continuity principles, it is expected that the leakage rate is consistent at any given radial direction and can be expressed by the below given relationship.

(8) $$q=\frac{h^{3}r}{12\eta p_{a}}\int_{0}^{2\pi }p\frac{\partial p}{\partial r}d\theta ,$$

The following dimensionless attributes were defined in order to investigate the performance of the seal.

(9) $$R=\frac{r}{r_{i}},P=\frac{p}{p_{a}},H=\frac{h}{c},$$

In turn, the dimensionless form of the Reynolds governing equation was obtained as shown below.

(10) $$\frac{\partial }{R\partial \theta }\left(\frac{H^{3}}{R}\frac{\partial P^{2}}{\partial \theta }\right)+\frac{\partial }{\partial R}\left(H^{3}\frac{\partial P^{2}}{\partial R}\right)=2\Lambda \frac{\partial \left(PH\right)}{\partial \theta },$$

where Λ is refers to the gas compression number, and its expression is presented as follows.

(11) $$\Lambda =\frac{6\mu \omega r_{i}^{2}}{p_{i}c^{2}},$$

where *μ* is the gas viscosity and *ω* is the rotational speed.

Substitute Equations (6) and (7), then dimensionless open force *F_o_* and dimensionless leakage *Q* can be obtained as follows.

(12) $$F_{o}=\frac{4\bar{F_{o}}}{d_{i}^{2}p_{a}},$$

(13) $$Q=\frac{12q\eta }{c^{3}p_{a}^{}}.$$

### 2.3. Calculation Method

The shape of the textured gas face mechanical seals employed for the intended analysis are provided in Figure 1. Unless otherwise specified, the main calculation parameters are as follows. The outer radius of work face was *r_o_* = 32 mm, the inside radius was *r_i_* = 24 mm, the textured diameter was *r_g_* = 27.5 mm, the periodic unite number in rotating directions was *N* = 120, the dimple number in radial directions was *n_r_* = 7, the inclination angle was *α* = 50°, the minor axis radius of ellipse was *a* = 150 μm, the slender ratio was *γ* = 4, the inlet groove depth was *h_g_* = 5 μm, and the dimple depth was *h_d_* = 5 μm.

In this research, finite difference method was used to conduct numerical analysis of the sealing performance. The convergence and mesh density of the program are presented below.

(1) Program convergence

**Figure 2:**
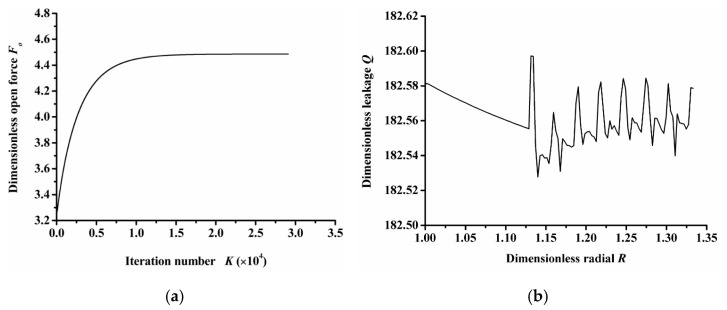
Program convergence verification: (**a**) convergence curve of opening force, (**b**) leakage rate along the radius.

Figure 2a shows the convergence curve of the opening force calculated through the finite difference method. As the iteration number increases, the opening force tends to be stable, which indicates that the numerical calculation method has good convergence and reliability.

According to the principle of flow conservation, the integral of radial flow through any radius is the same under steady state, that is, the calculated results should satisfy the law of mass conservation. Figure 2b shows the leakage rate of the sealing end face along the radius direction. The figure shows that the maximum leakage rate of the seal face along the radius direction was only 0.04% larger than the minimum value, indicating that the numerical calculation method meets the basic flow conservation principle of the Reynolds equation, which can ensure the rationality of the leakage rate calculation analysis.

(2) Mesh density

When the finite difference method is used, the mesh density affects the calculation’s accuracy and efficiency. Too high a mesh density wastes calculation time, but too low a mesh density cannot guarantee calculation accuracy. Therefore, reasonable mesh density should be selected for numerical analysis.

**Figure 3:**
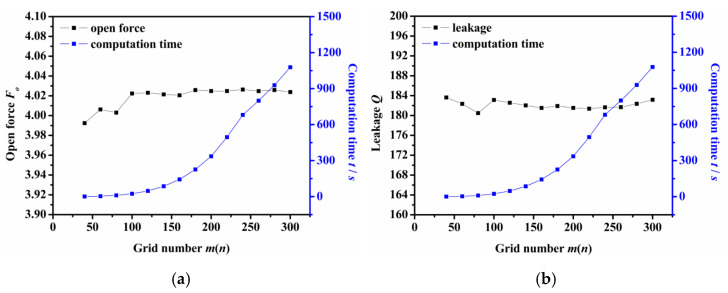
The influence of grid number on the calculation result: (**a**) opening force, (**b**) leakage rate.

Figure 3 shows the impact of grid number on the computation time and the calculation results of the opening force and leakage rate. Here, *m* is defined as the grid number along the circumferential direction, and *n* is the grid number along the radial direction. As the figure shows, the computation time increases as the grid numbers increases. When the grid number *m* × *n* was equal to or greater than 100 × 100, the calculated values of opening force and leakage rate remained almost unchanged with the difference between the maximum value and the minimum values were 0.14% and 0.98%, respectively. Therefore, the grid number can be selected as *m* × *n* = 100 × 100 to give consideration to the accuracy and efficiency of numerical analysis.

## 3. Results and Discussion

### 3.1. Influence of Operation Parameters

(1) Rotation speed

**Figure 4:**
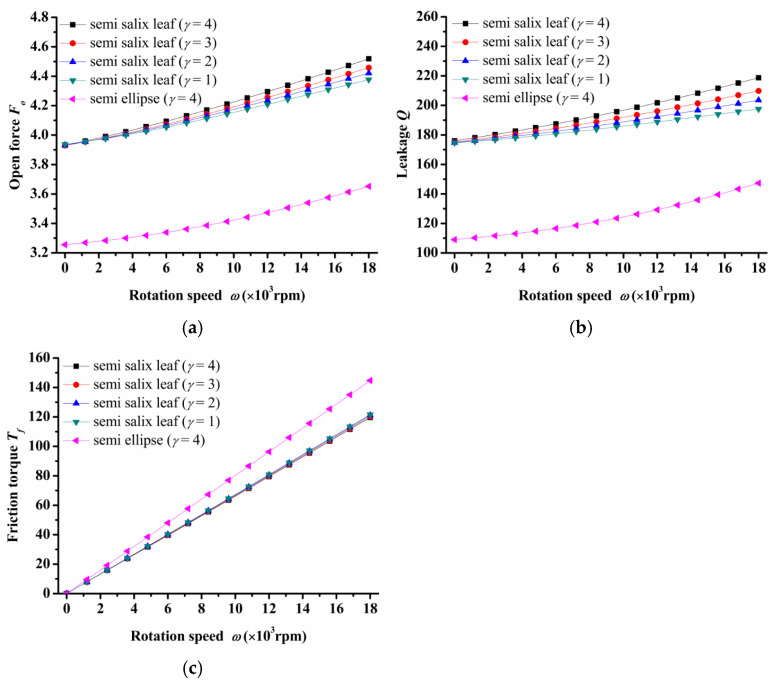
Influence of rotation speed on seal performance: (**a**) opening force, (**b**) leakage, (**c**) friction torque.

The influence of rotational speed on seal performance is depicted in Figure 4. As Figure 4a shows, the open force increased greatly as rotation speed increased for both semi salix leaf textured surfaces and the semi ellipse textured surface. This means that the seal faces had excellent opening behavior, which could decrease contact friction and wear during start-up as much as possible. Meanwhile, slender ratio *γ* influenced the open force significantly. The open force increased as the slender ratio increased, especially at higher speeds. All semi salix leaf textured surfaces had better open performance than the semi ellipse textured surface, which means that the inlet groove plays an important role in improving open performance and decreasing contact friction during the start-up stage. The leakage also increased as rotation speed increased and showed the same trend as the open force (Figure 4b). Figure 4c presents the friction torque of the seal surface. It can be seen that the higher the speed, the more obvious the improvement effect on friction torque. When rotation speed was 18,000 rpm, the open force, leakage, and friction torque of the semi salix leaf textured seal (*γ* = 4) was about 23% higher, 32% higher, and 22% lower, respectively, than that of the semi ellipse textured seal.

(2) Seal pressure

**Figure 5:**
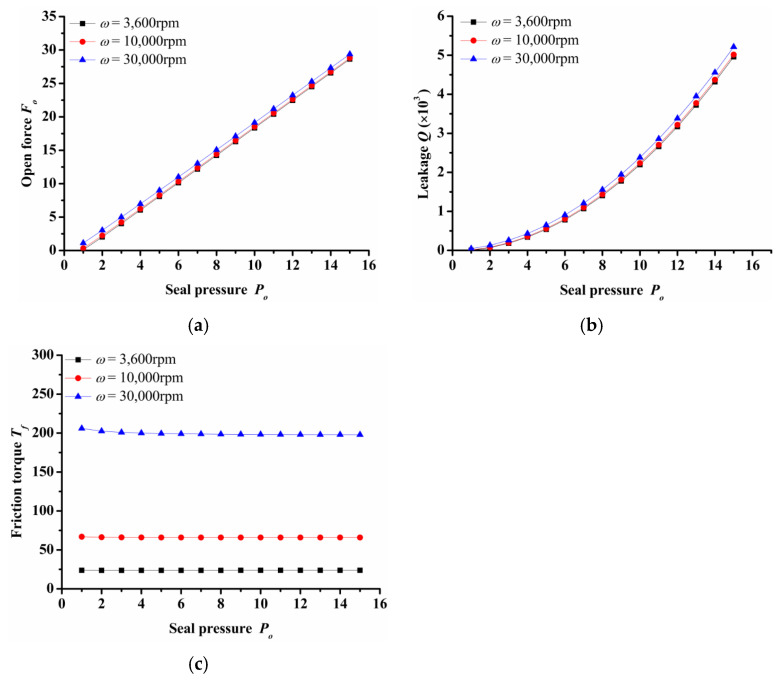
Influence of seal pressure on seal performance: (**a**) opening force, (**b**) leakage, (**c**) friction torque.

Figure 5 shows the impact of seal pressure over seal open force, leakage and friction torque under various rotation speed. It can be seen that both open force and leakage increased rapidly with the increasing seal pressure. The open force and leakage at *P_o_* = 16 was about 10 and 28 times respectively that of *P_o_* = 2 in the case of *ω* = 30,000 rpm. Both the increase in open force and leakage were mainly due to the increase in hydrostatic effect. In addition, the seal pressure had little influence on the friction torque. With the increase in pressure, the friction torque remained almost constant. However, when the rotation speed increased from 3600 rpm to 30,000 rpm, the friction torque increased about 10 times. This showed that the seal had a good hydrostatic pressure effect.

(3) Seal clearance

**Figure 6:**
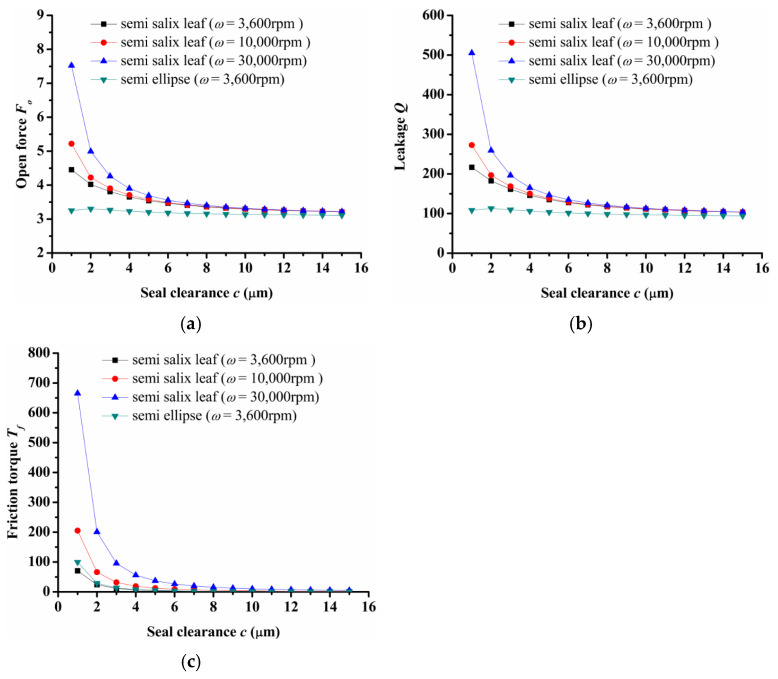
Influence of seal clearance on seal performance: (**a**) opening force, (**b**) leakage, (**c**) friction torque.

Figure 6 shows the seal clearance influence on seal performance. For a semi salix leaf textured seal, the open force, leakage, and friction torque decreased sharply as seal clearance increased and then almost remained constant when the seal clearance was larger than 8 μm. Meanwhile, the open force, leakage, and friction torque increased as rotation speed increased. It can be deduced that the open force, leakage, and friction torque with the rotation speed of *ω* = 30,000 rpm were about 68%, 136%, and 861% larger than the results when the rotation speed was 3600 when the seal clearance was 1 μm. However, for the semi ellipse textured seal without an inlet groove, the open force and leakage had no obvious change with the increasing seal clearance. When the rotation speed was 3600 rpm and the seal clearance was 1 μm, the open force and leakage were 26% and 49% lower than that of the semi salix leaf textured seal. The changing of friction torque showed the same trend as the semi salix leaf textured seal, but the value was larger under the same rotation speed of 3600 rpm. This means that the inlet groove plays a key role in reducing friction torque.

### 3.2. Influence of Texturing Parameters

(1) Inclination angle

**Figure 7:**
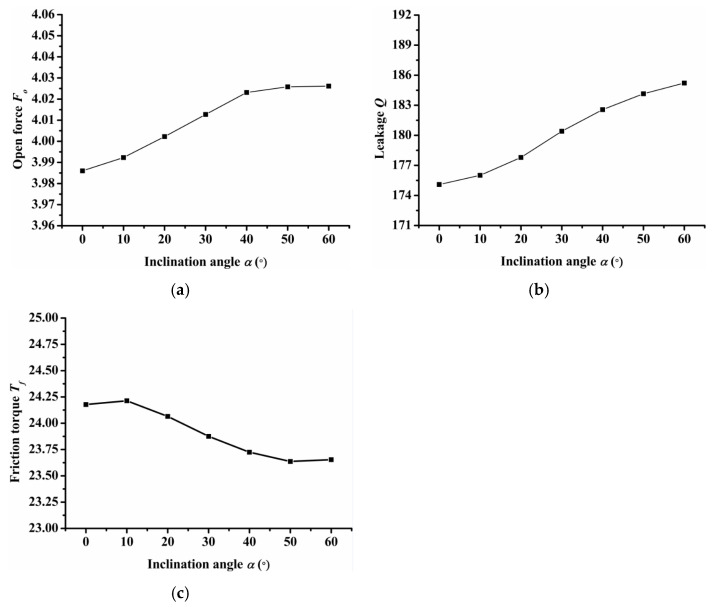
Influence of inclination on seal performance: (**a**) opening force, (**b**) leakage, (**c**) friction torque.

Figure 7 shows the influence of inclination angle on seal performance. It should be noted that when the angle of inclination is greater than 60 degrees, the dimples will connect. Therefore, only the seal performance of the inclination angle from 0 to 60 degrees was calculated. As Figure 7a shows, the open force increased as inclination angle increased and reached its maximum value when the inclination angle was equal to 50°. The leakage increased as the inclination angle increased, as shown in Figure 7b. When the angle of inclination was 50°, the friction torque reached the minimum value as shown in Figure 7c. Therefore, when the inclination angle was 50°, the open force was the largest and the friction torque was low, which would obtain a better seal performance when the inclination angle was 50°.

(2) Radial proportion of inlet groove

**Figure 8:**
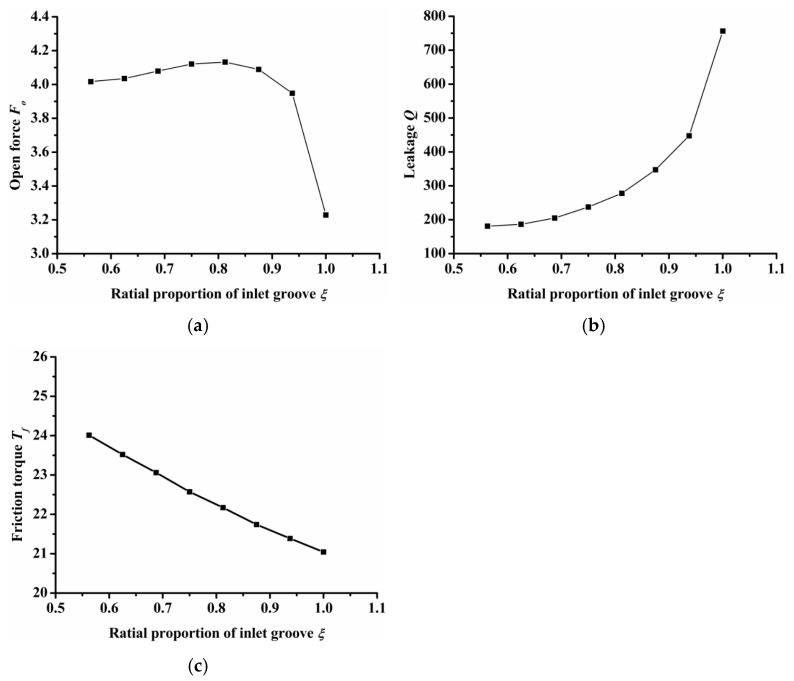
Influence of radial proportion of inlet groove on seal performance: (**a**) opening force, (**b**) leakage, (**c**) friction torque.

Figure 8 shows the influence of the radial proportion of inlet groove on open force, leakage, and friction torque. Here, the dimple number was constant at 7. When the radial proportion of the inlet groove was equal to 1, it indicates that the inner and outer diameters of the seal ring connect through the inlet groove. As shown in Figure 7a, the open force first increased and then decreased as the radial proportion of the inlet groove increased. When the radial proportion of the inlet groove was 0.8, the open force reached the maximum value. When the radial proportion of inlet groove was 1, the open force was the minimum, which was 23% lower than the maximum open force. This was because when the ratio was 1, the inner and outer diameters were connected and the high pressure gas leaked directly through the inlet groove from the outside to the inside diameter without the blocking effect of the seal dam, making the gas leak quickly. The gas cannot be squeezed by the seal dam, leading to a reduced open force. As the radial proportion of the inlet groove increased, the gas leakage resistance decreased, so the leakage increased. The gas quantity between the seal faces increased and the friction torque decreased.

(3) Dimple numbers

**Figure 9:**
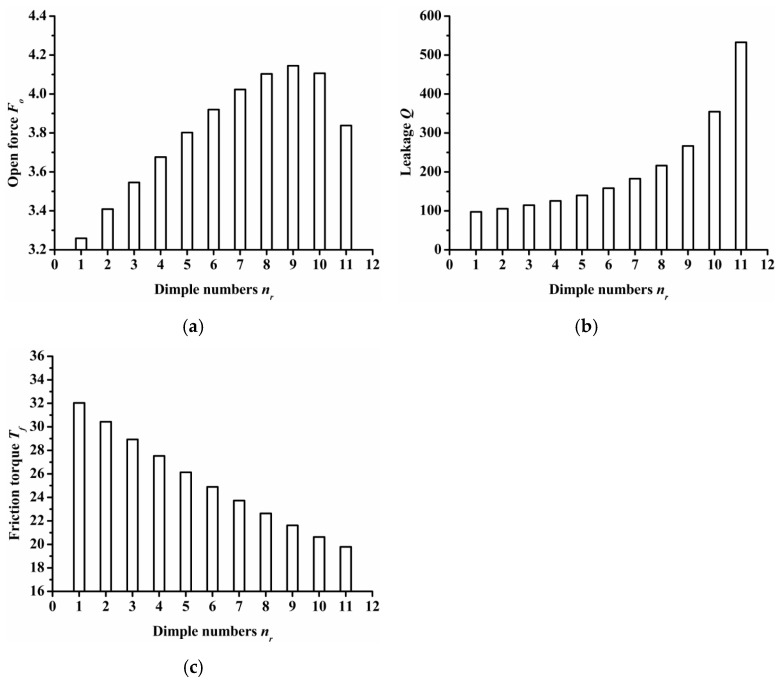
Influence of dimple numbers on seal performance: (**a**) opening force, (**b**) leakage, (**c**) friction torque.

The impact of dimple numbers on seal performance is summarized in Figure 9. For seal surface with various dimple numbers, the center distance between the semi ellipse dimples remained constant at 0.7 mm. Therefore, as the dimple number increases, the length of the inlet groove also increased as the results demonstrated that as the dimple number increased, the open force increased at first and then decreased, and reached its maximum value when the dimple number was 9. Obviously, the resistance of gas flowing from the outside radius to the inside radius decreased as the dimple number increased, so the leakage was increased and the friction torque decreased.

(4) Inlet groove and dimple depth

**Figure 10:**
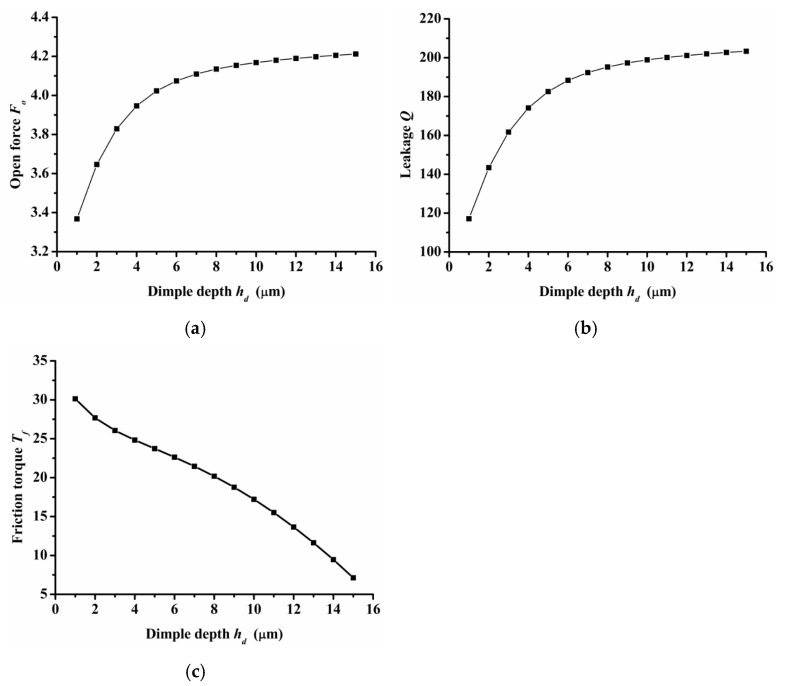
Influence of inlet groove and dimple depth on seal performance: (**a**) opening force, (**b**) leakage, (**c**) friction torque.

Figure 10 shows the performance of open force, leakage, and friction torque under various inlet groove dimple depths. During research, the depth of the inlet groove always remained the same as the depth of dimples. An important result was that both the open force and leakage increased as the dimple depth increased, but the gradient decreased. When the dimple depth increased from 1 μm to 6 μm, the open force and leakage increased about 17.4% and 3.4%, respectively. However, when the dimple depth increased from 6 μm to 15 μm, the open force and leakage increased about 62% and 7.7%, respectively. The friction torque decreased obviously as the dimple depth increased.

(5) Inlet groove angle

**Figure 11:**
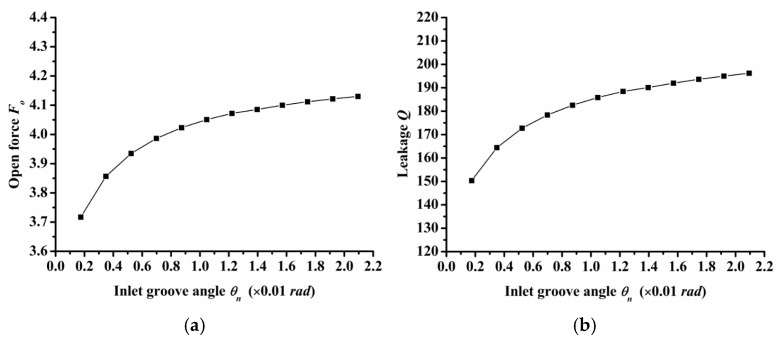

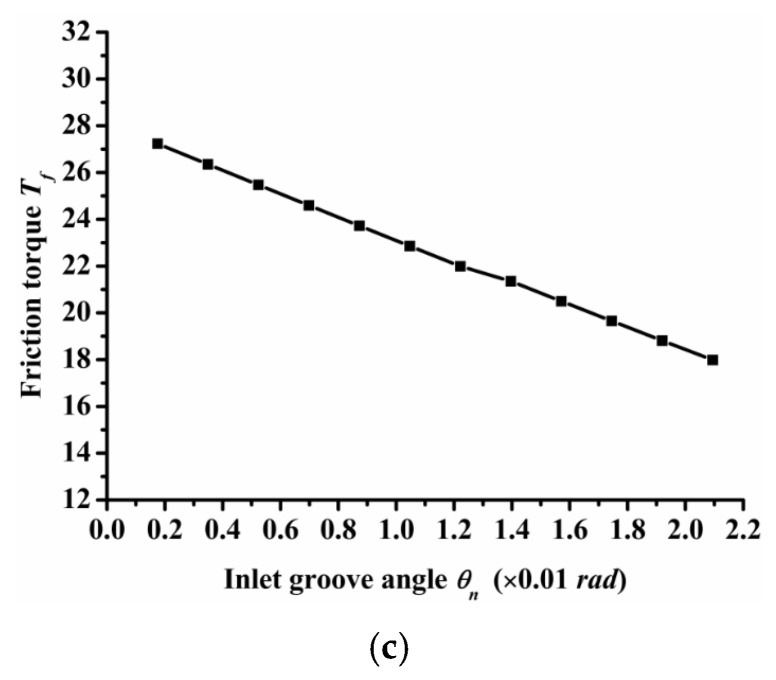
Influence of inlet groove angle on seal performance: (**a**) opening force, (**b**) leakage, (**c**) friction torque.

Inlet groove angle reflects the width of the inlet groove. The greater the angle, the greater the width of the inlet groove. Figure 11 shows the influence of inlet groove angle on open force, leakage, and friction torque. As the figure shows, open force and leakage increased as the inlet groove angle increased, but the friction torque decreased as the inlet groove angle increased. The changing trend of seal performance with inlet groove angle was consistent with that of seal performance with inlet and dimple depth. This is because the three parameters of inlet depth, dimple depth, and inlet angle can increase the amount of air intake, which will increase the open force, leakage, and decrease friction torque.

## 4. Conclusions

This article introduced and developed a novel gas mechanical face seal design with semi salix leaf textures to improve the hydrostatic and hydrodynamic performance of the gas face seal. The theoretical analysis model was established, then a series of parametric investigations were performed. From the above discussions, the following key conclusions are provided.

1. The semi salix leaf textured gas face seal had larger hydrostatic and hydrodynamic effects than the semi ellipse textured seal because of the effect of the inlet groove. All semi salix leaf textured surfaces had better open performance than the semi ellipse textured surface, which means that the inlet groove plays an important role in improving open performance and consequently decreasing the contact friction during the start-up stage. When the rotation speed was 18,000 rpm, the open force and friction torque of the semi salix leaf textured seal (*γ* = 4) was about 23% higher and 22% lower, respectively, than that of the semi ellipse textured seal.
2. The operation parameters greatly influenced the seal performance of the semi salix leaf textured gas face seal. The open force, leakage, and friction torque increased as the rotation speed increased. This means that the seal faces had excellent opening behavior, which could decrease contact friction and wear during start-up as much as possible. Both open force and leakage increased rapidly with the increase in seal pressure, which was mainly due to the increase in the hydrostatic effect. In addition, the seal pressure had little influence on the friction torque. With the increase in pressure, the friction torque remained almost constant. The open force, leakage, and friction torque decreased sharply as the seal clearance increased, and then almost remained constant when the seal clearance was larger than 8 μm.
3. The texturing parameters also influenced the seal performance of the semi salix leaf textured gas face seal. The open force increased as the slender ratio and inclination angle increased. When the inclination angle was 50°, the open force was the largest and the friction torque was low. As the radial proportion of the inlet groove increased, the open force first increased and then decreased. When the radial proportion of the inlet groove was 0.8, the open force reached the maximum value. As the dimple number increased, the open force increased at first and then decreased and reached its maximum value when the dimple number was 9. As the parameters of inlet depth, dimple depth, and inlet width increased, the amount of air intake increased, which will increase the open force, leakage, and decrease the friction torque.

This article introduced the theoretical analysis of the gas mechanical face seal with semi salix leaf textures. Next, it is necessary to carry out relevant experimental studies to advance the application of this seal face.

## Data Availability

The data are not publicly available as the work is ongoing.
